# High-throughput screening for in planta characterization of VOC biosynthetic genes by PTR-ToF-MS

**DOI:** 10.1007/s10265-019-01149-z

**Published:** 2019-11-07

**Authors:** Mingai Li, Luca Cappellin, Jia Xu, Franco Biasioli, Claudio Varotto

**Affiliations:** 1 Department of Biodiversity and Molecular Ecology, Research and Innovation Centre, Fondazione Edmund Mach, 38010 San Michele all’ Adige, TN Italy; 2 Dipartimento di Scienze Chimiche, Università degli Studi di Padova, Via Marzolo 1, 35121 Padua, Italy; 3 Dipartimento di Biologia, Università di Padova, Viale G. Colombo 3, 35121 Padua, Italy; 4 Department of Food Quality and Nutrition, Research and Innovation Centre, Fondazione Edmund Mach, 38010 San Michele all’ Adige, TN Italy

**Keywords:** Isoprene, Genetic screening, *Arabidopsis thaliana*, PTR-MS, Time-of-flight, VOCs

## Abstract

Functional characterization of plant volatile organic compound (VOC) biosynthetic genes and elucidation of the biological function of their products often involve the screening of large numbers of plants from either independent transformation events or mapping populations. The low time resolution of standard gas chromatographic methods, however, represents a major bottleneck for in planta genetic characterization of VOC biosynthetic genes. Here we present a fast and highly-sensitive method for the high-throughput characterization of VOC emission levels/patterns by coupling a Proton Transfer Reaction Time-of-Flight Mass Spectrometer to an autosampler for automation of sample measurement. With this system more than 700 samples per day can be screened, detecting for each sample hundreds of spectrometric peaks in the *m/z* 15–300 range. As a case study, we report the characterization of VOC emissions from 116 independent *Arabidopsis thaliana* lines transformed with a putative isoprene synthase gene, confirming its function also when fused to a C-terminal 3×FLAG tag. We demonstrate that the method is more reliable than conventional characterization of transgene expression for the identification of the most highly isoprene-emitting lines. The throughput of this VOC screening method exceeds that of existing alternatives, potentially allowing its application to reverse and forward genetic screenings of genes contributing to VOC emission, constituting a powerful tool for the functional characterization of VOC biosynthetic genes and elucidation of the biological functions of their products directly in planta.

**Electronic supplementary material:**

The online version of this article (10.1007/s10265-019-01149-z) contains supplementary material, which is available to authorized users.

## Introduction

Volatile organic compounds (VOCs) represent a relatively wide class of plant secondary metabolites. Their low molecular weight and high vapour pressure at ambient conditions allow them to freely exit through cellular membranes and reach the surrounding environment (Pichersky et al. 2006). More than 1,700 different VOCs have been detected and identified from emissions of plants belonging to 90 plant families widely distributed across land plant phylogeny (Knudsen et al. 2006). The fundamental roles of VOC emission in plants have been widely demonstrated. The first role elucidated for VOCs is the response of plants to abiotic stresses such as temperature, light and elevated ozone concentrations (Loreto and Schnitzler 2010; Vickers et al. 2009). More recent studies in the field of chemical ecology emphasize the crucial roles of VOCs as signaling compounds in plant–plant interactions (Baldwin et al. 2006), plant-pollinator communications (Raguso 2008) and plant defense (Hare 2011).

However, by contrast to the conserved function in abiotic stress responses, the role of VOCs in plant defense and reproduction is species-specific (Dudareva et al. 2013). There is an increasing evidence suggesting that the biosynthesis of VOCs, especially those constitutively emitted, is primarily regulated at the level of gene expression (Muhlemann et al. 2012). The discovery of genes linked to VOC emissions and the capability of manipulating emissions via genetic engineering/editing, on the one side, facilitate fundamental studies on the specific roles of VOCs in plant species (Dudareva et al. 2013). On the other side, these approaches may contribute to applications aimed to improve the natural defenses of crop plants, thus boosting the development of more environmentally friendly pest control strategies (Kos et al. 2013). Genetic manipulation of plant VOC emission is currently constrained by the limited understanding of the genetic control of VOC biosynthetic pathways (Dudareva et al. 2013). In addition, the high structural plasticity of VOC biosynthetic enzymes often makes a priori prediction of the enzymatic activity difficult if not impossible based on sequence homology only (Alquézar et al. 2017). Even though expression in bacterial or yeast cells is the fastest method to characterize VOC biosynthetic enzymes due to much shorter generation time, their expression in closely related model plants (e.g. *Arabidopsis thaliana* (L.) Heynh., *Nicotiana benthamiana* Domin for dicots or *Brachypodium distachyon* Roem. & Schult. for monocots) has the important advantage of providing a closer cellular environment where to characterize enzyme’s activity. Emerging evidences, in fact, point to the fact that subcellular localization, post-translational modifications and the levels of biosynthetic precursors in addition to enzyme promiscuity may affect the in vivo pattern of volatiles produced (Johnson et al. 2019; Pazouki and Niinemets 2016). In-planta characterization of VOC biosynthetic enzymes is, therefore, increasingly used, in addition or alternative to microbial production and in vitro assays, to elucidate the biological function of the produced VOCs (e.g. Aharoni et al. 2003; Gao et al. 2018; Salvagnin et al. 2016). In case soluble expression of VOC biosynthetic enzymes in *Escherichia coli* is not possible, *in*-*planta* overexpression represents one of the best alternatives or sometimes the only viable one (e.g. Li et al. 2017).

As compared with bacterial systems, however, a current bottleneck in plant transformation is the wide variation of expression levels in different transgenic lines (Afolabi et al. 2004; Klimaszewska et al. 2003; Kohli et al. 2006), which in turn requires the screening of large numbers of independent lines for transgene expression. Usually this is relatively work- and time-intensive as it first requires extracting RNA from a sufficient number of putative transformants, characterizing transgene expression levels by qRT-PCR and finally measuring the emission levels (Loivamäki et al. 2007; Sasaki et al. 2005). Selection of transgenic lines by direct measurement of VOC emissions provides an alternative method to identify the subset of transgenic lines with the desired VOC emission levels (e.g. the most highly emitting lines or a subset of lines with different emission levels to dissect the biological role of VOC(s) emission). For VOC emission measurements, the gold standard is represented by gas chromatographic (GC) columns coupled to adsorption–desorption devices. However, GC techniques, despite their great analytical power, suffer from limited time resolution—typically a single measurement takes from several minutes for fast GC columns to an hour for standard columns (Lacko et al. 2019).

Proton transfer reaction–mass spectrometry (PTR–MS) is a direct injection—mass spectrometric method allowing real-time monitoring of most VOCs with extremely low detection limits, typically in the low parts per trillion by volume (ppt_v_) range and, in some cases, even in the high parts per quadrillion by volume (ppq_v_) (Jordan et al. 2009a). Originally, PTR-MS instruments were equipped with a quadrupole mass spectrometer (Lindinger et al. 1998) providing good sensitivity and response time but a mass resolution limited to the nominal mass. PTR-MS has then been coupled with ion trap (Mielke et al. 2008; Prazeller et al. 2003) and time-of-flight (ToF) mass analysers (Graus et al. 2010; Jordan et al. 2009b). The latter provides higher time (20 Hz) and mass (*m*/∆*m* = 7 000) resolution and a wider mass range (Jordan et al. 2009b). Thanks to its high time resolution, high sensitivity and non-invasive detection properties, PTR-MS has cutting-edge applications in many fields including plant biology, environmental science, food science and technology and medicine (Blake et al. 2009).

Until now, however, PTR-MS has found limited application in genetic studies, except for fruit VOCs (Cappellin et al. 2015; Costa et al. 2013), due to its still limited throughput. Recently, an in vivo VOC phenotyping platform based on multiple cuvettes for whole plants was reported, which attains medium–high throughput (10–20 plants per hour; (Niederbacher et al. 2015), and constitute an important first step towards the throughput required by genetic screenings. In the present work we used a high-throughput system developed in-house for the investigation of VOC emission from genetically engineered *A. thaliana* plants using detached leaves. This is achieved by coupling a PTR-ToF-MS to an autosampler controlling conditions of single leaves and allowing VOC emissions from more than 30 plants per hour or about 700 plants per day. In this case-study we show that the approach is very effective in the screening of *A. thaliana* transgenic lines overexpressing a putative isoprene synthase gene from *Arundo plinii* Turra, allowing to reliably, rapidly and cheaply identify the lines with highest isoprene emission in a large number of independent transformation events.

## Materials and methods

### Plant material and growth conditions

Both wild-type Col-0 and transgenic Arabidopsis plants were grown at 23 °C in a growth chamber under standard long-day conditions (16 h light/8 h dark) at light intensity 120 ± 10 µmol photons m^−2^ s^−1^ and 40% relative humidity.

### *A. plinii IspS* cloning and transformation

The primers AplIspS_For (5′-CACCATGGCAATGGCTACCTGTAGT-3′) and AplIspS_Rev (5′-GGTACCAAATATACATGGTTCCAAGAAAAGC-3′) were used to amplify from leaf cDNAs the full-length CDS of the *IspS* gene of *Arundo donax* L. (GenBank accession number: ASF20076.1) and the putative *A. plinii* ortholog (GenBank accession number: MF685245) with an in frame KpnI site replacing the stop codon. The purified PCR products were directionally cloned into the Gateway vector pENTR/D-TOPO (Invitrogen) and verified by bidirectional Sanger sequencing. The 3xFLAG tag flanked by KpnI and AscI restriction sites was annealed from oligos 3×FLAG-For (5′-GGTACCTCGGATTATAAAGACCATGACGGAGACTATAAGGACCATGACCTCGACGCTGCAGCAGCGGATTATAAGGACGATGACGATAAGTGAGGCGCGCC-3′) and 3×FLAG-Rev (5′-GGCGCGCCTCACTTATCGTCATCGTCCTTATAATCCGCTGCTGCAGCGTCGAGGTCATGGTCCTTATAGTCTCCGTCATGGTCTTTATAATCCGAGGTACC-3′), phosphorylated, A-tailed, cloned into pGem-T and confirmed by bidirectional Sanger sequencing. After digestion with KpnI and AscI, the purified FLAG-tag fragment was subcloned in-frame at the C-TERM of each *IspS* CDS entry clone linearized with the same restriction enzymes. The resulting plasmids, pENTR_AdoIspS-3×Flag and pENTR_AplIspS-3×Flag plasmid sequences were recombined into the destination vector pK7WG2 through LR reaction with LR clonase II (Invitrogen). The final constructs were transformed into *Agrobacterium tumefaciens* strain GV3101-pMP90RK by electroporation and further transformed into *Arabidopsis thaliana* Col-0 ecotype by the floral dip method (Clough and Bent 1998). Transgenic plants were screened by plating sterilized seeds on solid Murashige and Skoog medium supplemented with 50 mg l^−1^ of kanamycin. Arabidopsis plants overexpressing the untagged *A. donax IspS* were described in (Li et al. 2017).

### Total RNA extraction and real-time PCR analysis

Total RNA from Col-0 wild-type and transgenic Arabidopsis plants was extracted with TRIzol Reagent (Invitrogen) according to manufacturer’s instructions. DNase treatment, cDNA synthesis from total RNA, and real-time PCR analyses were done as previously described (Fu et al. 2016). For real-time PCR analysis, reactions were performed with Platinum SYBR Green qPCR SuperMix-UDG (Invitrogen) in a Bio-Rad C1000 Thermal Cycler detection system using primer pairs AplIspS_RT_For: 5′-GAGGTTCCGTTGCATTTGAG-3′ and AplIspS_RT_Rev: 5′-CAAGAGCAACATCTGTCCAC-3′ for *AplIspS* as target and AtactII_RT_For: 5′-GCACCCTGTTCTTCTTACC-3′ and AtactII_RT_Rev: 5′-AACCCTCGTAGATTGGCACA-3′, for Arabidopsis *ActinII* as reference gene. All reactions were performed in triplicates and the ∆∆Ct method was used to calculate fold changes.

### Isoprene emission analysis by PTR-ToF–MS

A single, fully developed rosette leaf from 4-week old T1 transgenic plants was cut, weighed and transferred into 20 ml glass vials containing 300 µl distilled water and equipped with PTFE/silicone septa (Agilent; Cernusco sul Naviglio, Italy). The vials were left open for 30 min to remove green leaf volatiles. After sealing, the vials were incubated at 30 °C with photon flux density of l30 ± 10 µmol m^−2^ s^−1^ for 3 h in a growth chamber and loaded onto the temperature-controlled autosampler blocks.

During measurements 100 sccm of zero air was continuously injected into the vial through a needle heated to 40 °C and the outflow going through a second heated needle was delivered via Teflon fittings to the PTR-ToF-MS. Each measurement lasted for 30 s and vials were automatically switched using an adapted GC autosampler (MPS Multipurpose Sampler, GERSTEL; Mülheim an der Ruhr, Germany). Vials were measured in randomized order. One Col-0 leaf was used in each autosampler block of 32 vials as reference. Measurements were performed with a commercial PTR-TOF 8,000 apparatus from Ionicon Analytik GmbH (Innsbruck, Austria), equipped with a ToF from TofWerk AG (Thun, Switzerland). The reaction conditions in the drift tube were the following: 550 V drift voltage, 2.3 mbar drift pressure, 110 °C drift tube temperature, leading to an *E/N* ratio (*E* corresponds to the electric field strength and *N* to the gas number density) of about 140 Townsend (Td; 1 Td = 10^−17^ V cm^2^). Details on the instrument operating conditions can be found in (Cappellin et al. 2011a).

### Data analysis

#### Analysis of ToF spectra

PTR-ToF-MS spectra post-processing generally followed the methodology described by Cappellin et al. (2011a), with some modifications. First, the count losses related to the detector dead time was accounted for as in Cappellin et al. (2011b) using a correction based on Poisson statistics. After internal mass calibration, the achieved mass accuracy (greater than 0.001 Th) allowed determination of the sum formula of ions corresponding to the spectral peaks. Noise reduction, baseline removal and peak intensity extraction were performed using modified Gaussians to fit the peaks (Cappellin et al. 2011a). Absolute VOC concentrations in the sample headspace were calculated from peak intensities as previously described (Lindinger et al. 1998) and considering H_3_O^+^ as primary ion and the actual rate coefficient for ion–molecule reaction at the selected energetic conditions (Cappellin et al. 2012). Headspace concentrations were normalized by leaf fresh weight.

#### Statistical analysis

Welch one-way test at a significance level of *p* < 0.05 and the Bonferroni correction were employed to determine differences in VOC emission among transgenic lines. The normality assumption was checked by a Shapiro–Wilk test. Correlation analysis between isoprene emission and transgene expression was carried out by least square regression followed by bootstrap estimation of 95% confidence intervals (*N* = 1,999). All analyses were carried out using routines written in *R*.

## Results

### Screening system features exemplified by a case study

**Fig. 1:**
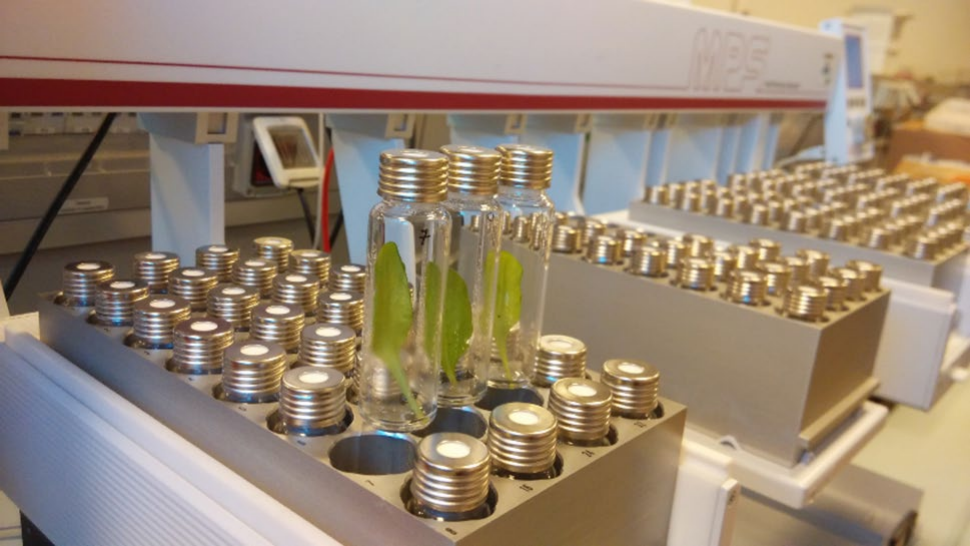
Autosampler used for leaf screening. Vials with single Arabidopsis leaves in the autosampler blocks

The autosampler integrated to PTR-ToF-MS is shown in Fig. 1. Sample preparation was intentionally kept down to a minimum, leveraging on the ability of the system to read with high sensitivity the head-space of single leaves sealed in semi-disposable vials with a total volume of 20 ml. In the case-study presented, addition of hydroponic medium to the tubes, leaf cutting, insertion in the tubes, and sealing of the vials usually takes no more than 2 h for a total of 160 samples, corresponding to 5 blocks of the autosampler. The system allows up to 15 h of unsupervised data acquisition. The costs of consumables for routine screening are mainly due to the Teflon seals (which are pierced by the autosampler upon measurement), as the glass vials and metal caps can be washed and re-used. All other costs for consumables (e.g. hydroponic medium and gases for PTR-ToF-MS operation) are negligible. Light intensity, temperature and incubation time were chosen based on literature (Loivamäki et al. 2007) to maximize isoprene emission from Arabidopsis leaves, but different conditions may be required by other VOC genes. In general, however, incubation times of up to 4–5 h are still compatible with completion of a round of screening within 24 h. The analytical protocol of the screening system was in fact optimized to maximize daily throughput without compromising on the range of measured ions, resulting in the ability to detect for each sample a total of 184 spectrometric peaks in the *m/z* 15–300 range while screening about 30 samples per hour or more than 700 samples per day.

To demonstrate the speed and sensitivity of the VOC screening platform obtained here we carried out as a case-study the characterization of isoprene emission from 116 independent Arabidopsis lines transformed with a putative isoprene synthase (*IspS*) gene of the rhizomatous grass *Arundo plinii*. In this way we aimed to achieve three main results: (1) to test whether C-terminal addition of the 3xFLAG tag to *IspS* CDS would be compatible with enzymatic activity, (2) to confirm in vivo isoprene emission from Arabidopsis transformed with the *A. plinii IspS* transgene, and (3) to select highly emitting lines for further studies.

## 3×FLAG-tagged *IspS* is compatible with enzymatic activity

**Fig. 2:**
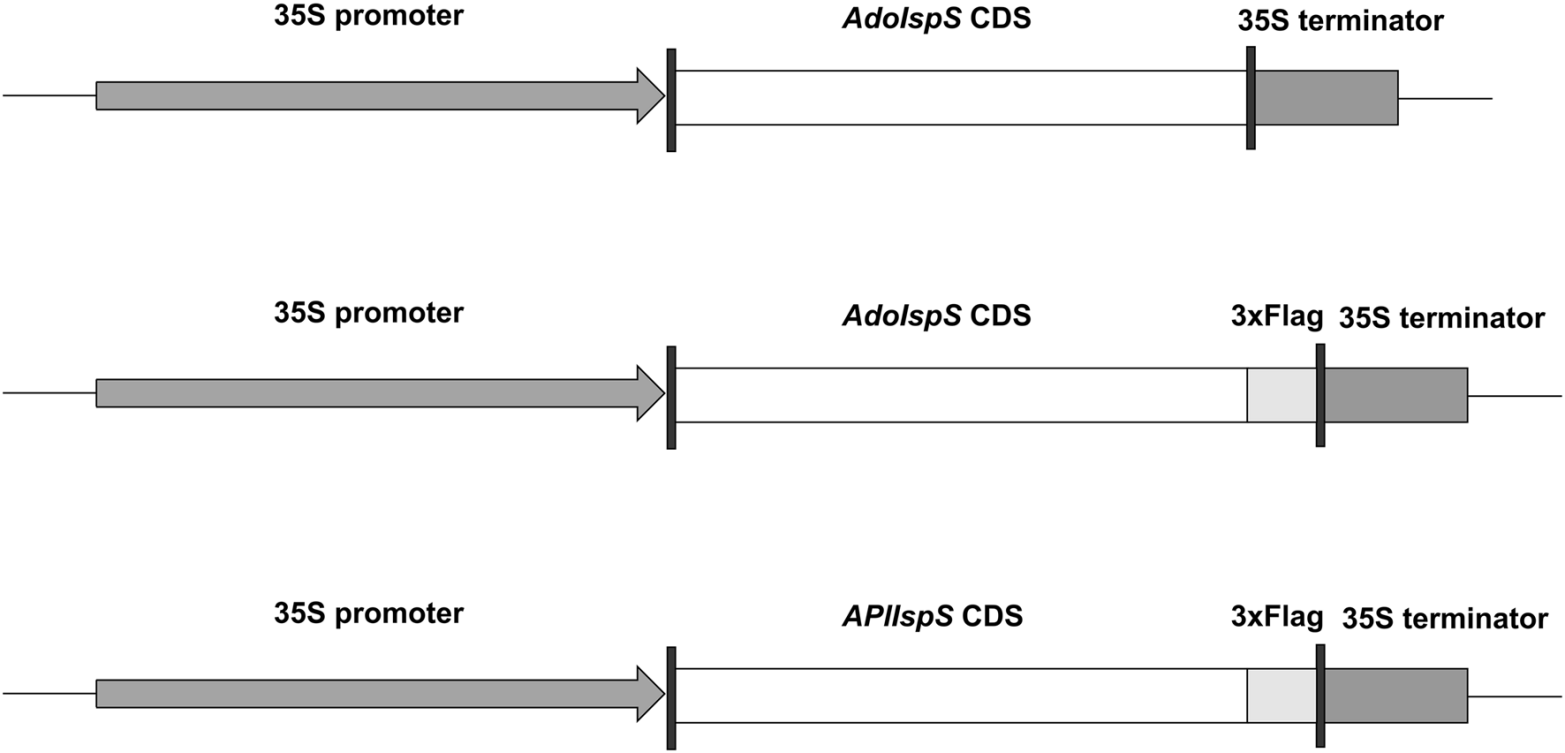
Constructs used for Arabidopsis transgenic lines. The full-length cDNA of *AdoIsps* or *AplIsps* fused with in-frame 3×Flag tag was driven by the 35S promoter from Cauliflower Mosaic Virus

Previous attempts to solubly express either the validated *A. donax* or the putative *A. plinii IspS* in *E. coli* failed (Li et al. 2017), thus we first tested whether an alternative system based on *in*-*planta* tagging could be established as a viable alternative for quantification and/or purification from highly emitting transgenic lines of Arabidopsis. C-terminal tagging of either *A. donax* or *A. plinii* were compatible with enzymatic activity as determined by measurement of the C_5_H_9_^+^ ion, representing the major product of the ion–molecule proton transfer reaction between H_3_O^+^ primary ions and isoprene (de Gouw et al. 2003), emitted from the respective Arabidopsis transgenic lines (Table 1, Fig. 2). The possible interference of alkyl fragments from other volatiles to the peak corresponding to the ion C_5_H_9_^+^ at *m/z* 69.069 was neglected. In fact, the parent ions for interfering volatiles at higher *m/z* values were several orders of magnitude less intense than the ion C_5_H_9_^+^ and therefore such potential interference can be considered minor and does not affect our conclusions. In the case of *A. donax IspS*, a transgenic line could be identified with amounts of the C_5_H_9_^+^ ion per unit of fresh weight comparable to those of the previously characterized untagged enzyme (Li et al. 2017; Table 1). As expected, the C_5_H_9_^+^ ion corresponding to isoprene was about 3–4 orders of magnitude more abundant among those differing between transgenic and untransformed Col-0 plants (Table 1; Online Resource 1). The other detected ions with differential emission corresponded to those reported for butanal-2-methyl (Mochalski et al. 2014); monoterpenes and their fragments (de Gouw et al. 2003; Maleknia et al. 2007).

**Table 1:** Summary of differences in VOCs emitted by Arabidopsis transgenic (*A. donax* tagged and untagged *IspS*) and control lines (Col-0)

*m/z* (Th)	Ion	Annotation	AoIspS_3xFLAG	AoIspS	Col-0	*p* value
53.0394	C_4_H_5_^+^	Unidentified	0.118 ± 0.026 **b**	0.101 ± 0.010 **b**	0.0026 ± 0.0014 **a**	1.6 × 10^−7^***
69.0683	C_5_H_9_^+^	Isoprene (de Gouw et al. 2003)	74 ± 20 **b**	71 ± 7 **b**	0.14 ± 0.04 **a**	7.0 × 10^−7^***
85.0648	C_5_H_9_O^+^	Butanal, 2-methyl (Mochalski et al. 2014)	0.033 ± 0.006 **c**	0.0221 ± 0.0019 **b**	0.0103 ± 0.0019 **a**	2.0 × 10^−6^***
95.0845	C_7_H_11_^+^	Monoterpene fragments (Maleknia et al. 2007)	0.0109 ± 0.0017**c**	0.0083 ± 0.0008 **b**	0.0052 ± 0.0007 **a**	1.8 × 10^−5^***
109.1021	C_8_H_13_^+^	Monoterpene fragments (Maleknia et al. 2007)	0.030 ± 0.007 **b**	0.0293 ± 0.0029**b**	0.009 ± 0.004 **a**	3.0 × 10^−5^***
137.1328	C_10_H_17_^+^	Monoterpenes (de Gouw et al. 2003)	0.065 ± 0.017 **b**	0.066 ± 0.008 **b**	0.015 ± 0.005 **a**	1.1 × 10^−5^***

Emissions of volatile compounds are expressed in terms of headspace concentration detected by PTR-ToF-MS analysis normalized by leaf fresh weight (ppb_v_ mg FW^−1^, or parts per billion by volume per mg fresh weight). Values for each genotype represent mean ± standard deviation. Welch one-way test at a significance level of *p* < 0.05 was employed to compute *p* values by comparing compounds. For each compound, values marked with the same letter (bold) do not significantly differ from each other. Asterisks indicate statistical significance after Bonferroni correction. Significance codes: 0 ‘***’ 0.001 ‘**’ 0.01 ‘*’ 0.05

### *AplISpS* is an isoprene synthase

Also, in the case of *A. plinii* putative *IspS*, tagging with the 3xFLAG was compatible with enzymatic activity as demonstrated by the identification of transgenic lines with amounts of the C_5_H_9_^+^ ion per unit of fresh weight comparable to those of the tagged *A. donax* enzyme. The deduced amino acid sequence of the putative *IspS* gene isolated from *A. plinii* is 98.8% identical to the one recently characterized from the biomass species *A. donax* and all the features demonstrated to be relevant for catalytic activity are conserved (Fig. S1) (Li et al. 2017). In addition, the majority of the six amino acid substitutions among enzymes are conservative. Consistently, VOC emission profile of *AplIspS_3×FLAG* lines displayed a very strong linear correlation with those of either tagged and untagged *AdoIspS* lines (*AplIspS_3×FLAG* vs. *AdoIspS_WT_79*: *R*^2^ = 0.999, *p*(uncorrelated) = 1.18 × 10^−288^; *AplIspS_3×FLAG* vs. *AdoIspS_3×FLAG_79*: *R*^2^ = 0.992, *p*(uncorrelated) = 5.93 × 10^−192^), but no significant correlation with Col-0 emission profile at *α* = 0.05 (*AplIspS_3×FLAG* vs. Col-0: *R*^2^ = 0.010, *p*(uncorrelated) = 0.18432). Taken together, these results confirm that the gene isolated from *A. plinii* is a functional *IspS* synthase.

### Identification of highly emitting *AplIspS_3×FLAG* transgenic lines

**Fig. 3:**
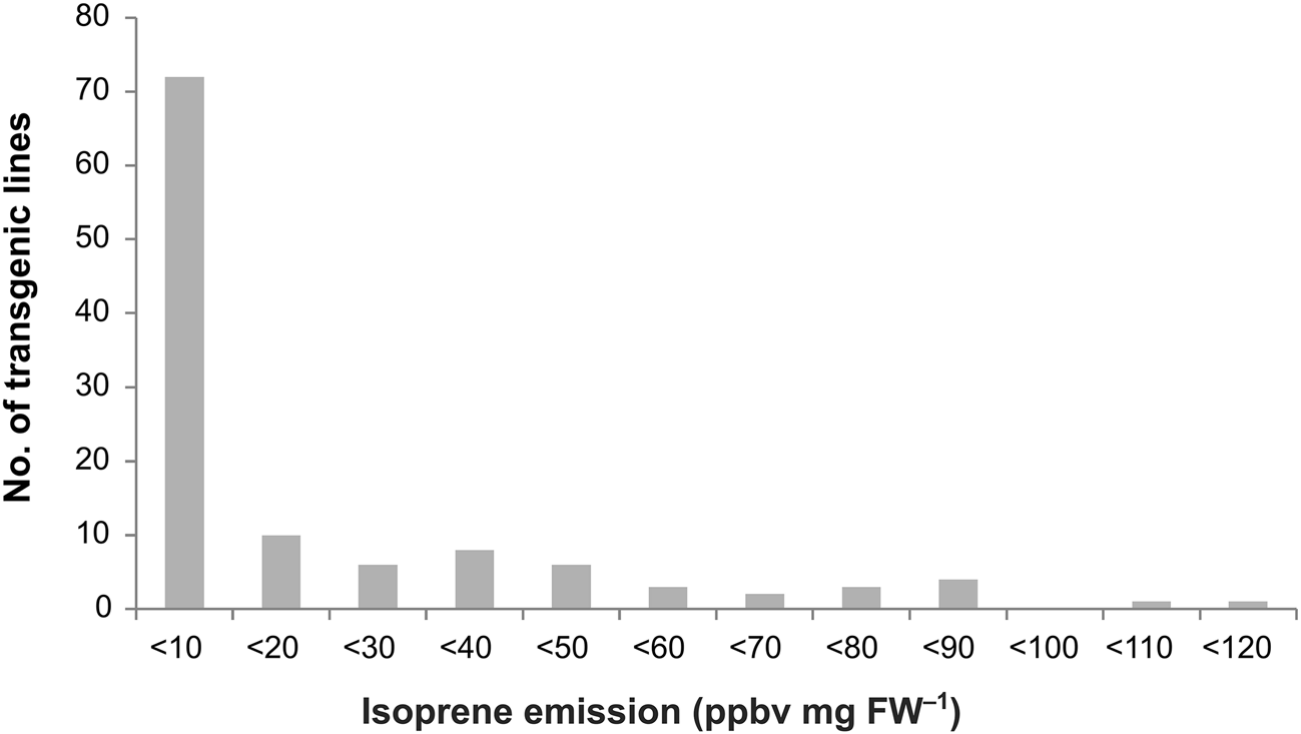
Characterization of Arabidopsis lines overexpressing the *AplIspS* gene. Distribution of isoprene emissions from 116 transgenic lines overexpressing the tagged *AplIspS*. *ppb*_*v*_ parts per billion by volume

Among the 116 independent *AplIspS_3×FLAG* transgenic lines analyzed (Fig. 2), the majority (*n* = 72) had isoprene emissions lower than 10 ppb_v_ mg FW^−1^ (Fig. 3; Online Resource1). In general, isoprene emission was not normally distributed across lines (Shapiro–Wilk test: *p*(normal) = 1.34 × 10^−13^), with strong skewing towards low emission values (median emission: 4.91 10 ppb_v_ mg FW^−1^; Skewness: 1.78; Kurtosis: 2.55; Fig. 3), consistent with positional effects due to integration in heterochromatic genome regions (Koncz et al. 1989). However, the top-emitting lines (*n* = 9) reached emission levels > 80 ppb_v_ mg FW^−1^ (Online Resource 1).

**Fig. 4:**
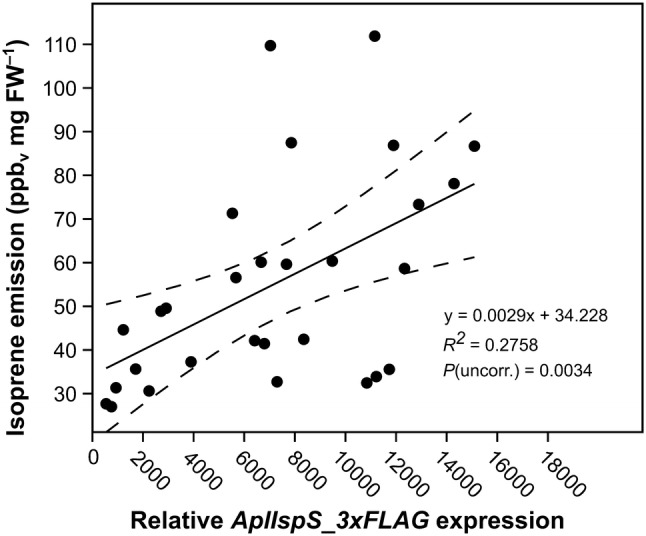
Correlation between IP emission and transcription of *AplIspS* gene in 29 transgenic Arabidopsis lines. Black dots correspond to each of the 29 lines analyzed. Blue lines represent 95% confidence intervals for the regression line shown in red

To test whether isoprene emission would correlate with transgene expression levels, we extracted total RNA from the top 30 emitting Arabidopsis lines, resulting in 29 successful cDNA preparations. Transgene expression levels were significantly but relatively weakly correlated with isoprene emission (*R*^2^ = 0.2758, *p*(uncorrelated) = 0.0034439; Fig. 4). Thus, lines with very similar expression can widely vary in terms of emission and, conversely, lines with similar emission can be characterized by variable transgene expression (Fig. 4).

## Discussion

High throughput, ease and low cost are very important features for any kind of screening to be feasible. As shown by the case-study presented, the method described here has all these features, and can thus be extremely useful for the screening and a general characterization of plant VOC biosynthetic genes overexpressed in model plant systems, because: (1) it identifies/quantifies the main candidate VOCs directly in living plant cells, thus in more realistic conditions than those reproduced in distantly heterologous systems like *Escherichia coli* or in in vitro enzymatic assays with purified proteins (Johnson et al. 2019; Pazouki and Niinemets 2016); (2) it has a throughput of several hundred samples per day and allows unsupervised overnight measurements; (3) it requires minimal hands-on time and costs in terms of preparation of the samples and operation; (4) the minimum sample amount is very small (one leaf or part of it), owing to the high sensitivity of the instrument (Jordan et al. 2009a) and (5) it is a green analytical technique as no solvents or spare parts are required besides the pierceable septa.

**Fig. 5:**
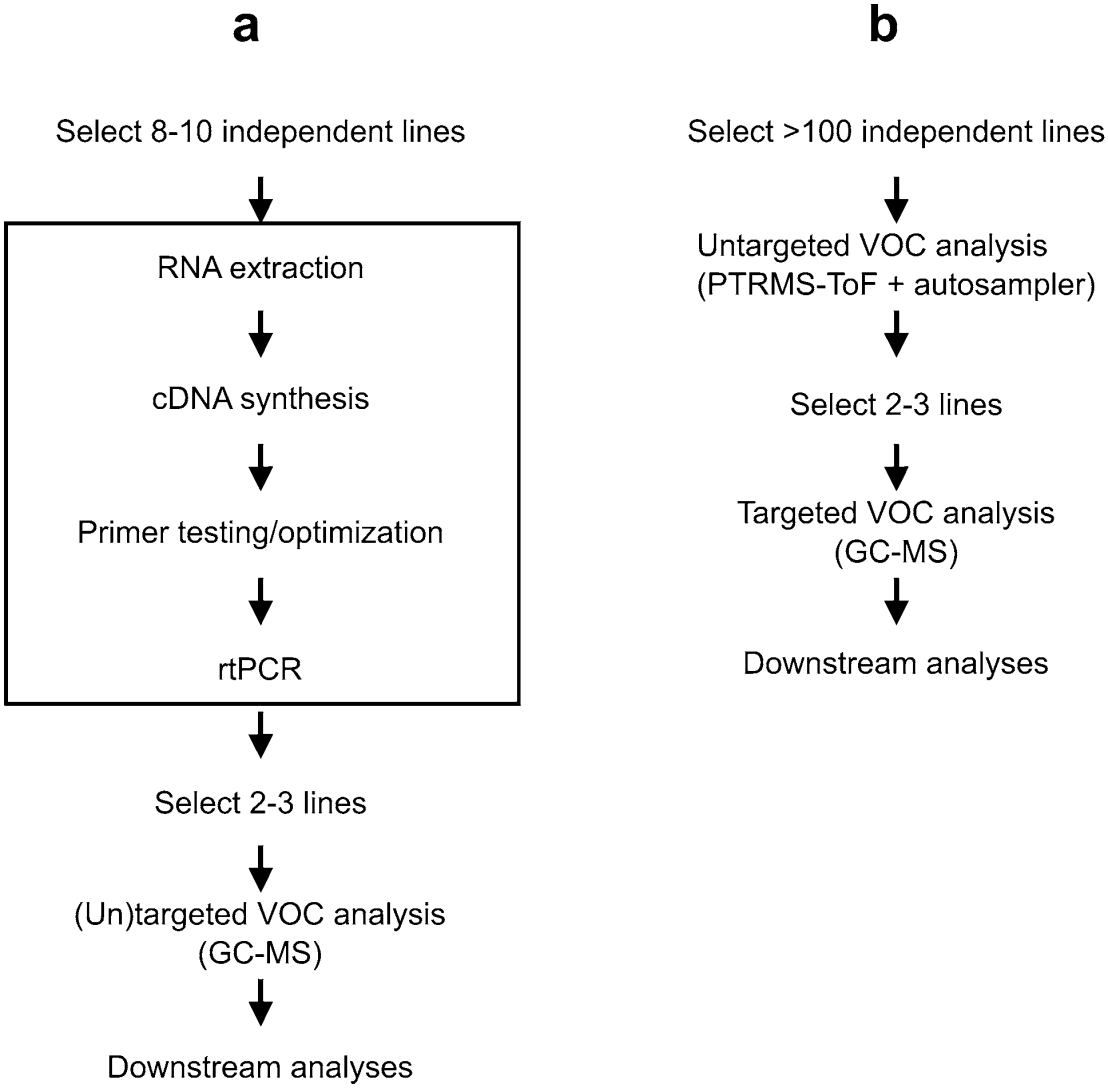
Comparison between screenings of candidate VOC biosynthetic genes. **a** A typical genetic screening and **b** the screening system described here for the characterization of a candidate VOC biosynthetic gene. The box indicates steps related to the characterization of transgene expression

As compared to a typical genetic approach, this method allows screening of much higher numbers of candidate transgenic lines and can further characterize in detail only those selected for the purpose of the screening (e.g. lines with maximal emission or lines representing the whole spectrum of emissions of a given VOC). This, for instance, has the advantage of minimizing the incidence of costs and work associated to characterization of transgene expression levels (Fig. 5; see boxed steps). In particular, identification of the most highly emitting lines can be particularly valuable in basic research to comparatively characterize in a common genetic background the effects of different VOCs on sometimes subtle plant fitness effects with respect to biotic and abiotic stresses (Brilli et al. 2019; Huang et al. 2013; Palmer-Young et al. 2015; Truong et al. 2014). From an applicative point of view the screening method presented here further allows identification of the lines with the highest emission of a specific VOC that guarantee the best compromise between stress tolerance and growth. It is worth noting that, our results demonstrate on a large scale that the expression level of the transgene is not a very good proxy of VOC emission, in line with previous observations (Loivamäki et al. 2007). Thus, direct VOC measurement with a high throughput method like the one described here is possibly the best option for the characterization of biological VOC gene functions *in planta*.

A limitation of the method is that it only provides an initial characterization of the VOC spectrum produced by the transgenic plants, but it cannot provide, in general, a conclusive compound identification as reliably as GC–MS (Majchrzak et al. 2018). In the simple case study carried out here this is not an issue, as the extremely high sequence similarity of the analyzed gene and the pattern of VOC emission nearly identical to that of the previously characterized *AdoIspS* (Li et al. 2017) rule out that *AplIspS* could have different functions. In general, however, if applied to the study of previously uncharacterized VOC biosynthetic genes with lower sequence identity [e.g. from lower plants; (Kumar et al. 2016)] or lacking reference emission patterns with extremely high linear correlation, the method requires the use of targeted GC–MS methods with appropriate standards to confirm the identity of the emitted VOCs (Matarese et al. 2014; Vrhovsek et al. 2014). This is particularly relevant, for instance, for families of isobaric compounds like monoterpenes, which cannot easily be discriminated on their fragmentation *m/z* ratios under standard ionization conditions (Tani et al. 2003). These limitations can be overcome by integration of further separation steps (Khomenko et al. 2017). On the other hand, already in its simplest configuration, the method can pinpoint the major ions and significantly narrow down the possible candidates associated to them, thus guiding and simplifying the GC–MS-based identification which can be performed on a small sample set.

Based on the results presented, the system described here cannot be, strictly speaking, defined as a phenotyping system (providing by definition non-destructive measures; Fahlgren et al. 2015), as all data refer to detached Arabidopsis leaves. However, the system is sensitive enough to be applied also non-destructively to Arabidopsis or other plant seedlings grown for up to 7–10 days in the measuring vials containing a small volume of hydroponic medium. It is, therefore, likely that in the future the system could be relatively easily adapted to the phenotyping of VOC profiles of plant species with small seedlings. As compared to the high-throughput plant phenotyping system developed by Schnitzler and colleagues (Niederbacher et al. 2015), the small and semi-disposable vials necessary to the autosampler, do not allow the possibility to monitor VOC emission of fully grown plants for long periods and with the precise environmental control provided by larger measuring cuvettes and highly controlled setups. On the other hand, this limitation is at least partly offset by the easier sample preparation/handling and the more than double throughput offered by our system (Niederbacher et al. 2015), which could thus find complementary applications in cases where the large number of samples and the rapidity of the screening are key features. For instance, the system described here could potentially be used at an advantage in several applications, like characterization of VOC emission under different nutritional conditions (Kusano et al. 2013), forward and reverse genetics screenings of highly divergent VOC biosynthetic genes from basal plant species (Kumar et al. 2016), screening of natural variation of VOC emissions from ecotypes or species (Defossez et al. 2018; Kergunteuil et al. 2019), and in other applications requiring high throughput.

## Electronic supplementary material

Below is the link to the electronic supplementary material.

Online Resource 1: VOC emissions of the genotypes analyzed in this study. (XLSX 230 kb)

Supplementary material 2 (PDF 707 kb)
