# Supplementary material for: High-throughput screening for in planta characterization of VOC biosynthetic genes by PTR-ToF-MS
Source: J Plant Res. 2019 Nov 7;133(1):123–31. doi: 10.1007/s10265-019-01149-z (PMC6946754; doi:10.1007/s10265-019-01149-z)
Supplement: Supplementary file 2 — Supplementary material 2 (PDF 707 kb) [file 10265_2019_1149_MOESM2_ESM.pdf]

High-throughput screening for *in planta* characterization of VOC biosynthetic genes by PTR-ToF-MS

Mingai Li[1,#], Luca Cappellin[1,2,#], Jia Xu[1,3], Franco Biasioli[4,*] and Claudio Varotto[1,*]

[1] Department of Biodiversity and Molecular Ecology, Research and Innovation Centre, Fondazione Edmund Mach, I-38010, San Michele all' Adige, TN, Italy
[2] Dipartimento di Scienze Chimiche, Università degli Studi di Padova, Via Marzolo 1, I-35121, Padova, Italy
[3] Dipartimento di Biologia, Università di Padova, Viale G. Colombo 3, I-35121, Padova, Italy
[4] Department of Food Quality and Nutrition, Research and Innovation Centre, Fondazione Edmund Mach, I-38010, San Michele all' Adige, TN, Italy
[#] These authors equally contributed to this work
[*] Corresponding author

ONLINE RESOURCE 2

```
                        10        20        30        40        50        60        70        80        90       100
                ....|....|....|....|....|....|....|....|....|....|....|....|....|....|....|....|....|....|....|....|
A. donax IspS   MAMATCSWPSPPVQLRVMASKQPQPEQRRSANYQPNAWDYSSLLSLKGRGNDQNLTNPSVFNKLKASVRDLLINKPEPSAKLRLIDTMQRLGISYHFEEE
A. plinii IspS  ........................................G.....................................................N.....

                       110       120       130       140       150       160       170       180       190       200
                ....|....|....|....|....|....|....|....|....|....|....|....|....|....|....|....|....|....|....|
A. donax IspS   ISAILSSISQESAKAPFMDDVASMALKFRMLRGNGFDVSTELLSSLLDGKNSFRTVPHSDIDGLLSVYEASYLAFHGEDMFDELRKNSASALKDLLPSMD
A. plinii IspS  ..................................................................................D.................

                       210       220       230       240       250       260       270       280       290       300
                ....|....|....|....|....|....|....|....|....|....|....|....|....|....|....|....|....|....|....|
A. donax IspS   SHMRSNVAYSLEVPLHLRAPRLEARWFIDHYERCMASDDLIIQFAKQDFNNVQSMHTQELAALTSWWTDVALGEKLTFARDRLIECFHYANGVMWEPNLA
A. plinii IspS  ...................................G................................................................

                       310       320       330       340       350       360       370       380       390       400
                ....|....|....|....|....|....|....|....|....|....|....|....|....|....|....|....|....|....|....|
A. donax IspS   KCREAITKAFALVVHLDDVYDVYGTLDELSLFTEAMGRWDVSASEALPEYMRAIYCTIINTSNEMADHVLREQGQSTQHLFHKGWHDLCKAFLVEAKWHY
A. plinii IspS  .............................................T......................................................

                       410       420       430       440       450       460       470       480       490       500
                ....|....|....|....|....|....|....|....|....|....|....|....|....|....|....|....|....|....|....|
A. donax IspS   GNYRPTLSEYLNNGWMSSSGPLLLLHAFPFLNEKFSTKHLEWLERYPRIVQSSSKIFRLCNDSATHSDELQRGDAPSSIAIYMLENNADEQESRAAMHEY
A. plinii IspS  ..................................I.................................................................

                       510       520       530       540       550       560
                ....|....|....|....|....|....|....|....|....|....|....|....|....
A. donax IspS   TIDIWKSVNEEAFCHHHYPKSFRKACLNLSRISHCIYQGGDGLGAPDDEKKKQIKELFLEPCIF
A. plinii IspS  ......................................................R.........
```

Fig. S1. Sequence alignment between *A. plinii* and *A. donax* proteins. The whole protein sequence deduced from the transcript of the novel *A. plinii IspS* gene was aligned to *A. donax* IspS (GenBank accession number ASF20076.1) with ClustalW. Dots indicate identical residues among proteins. The 16 amino acids shaded in green indicate the chloroplast targeting signal peptide; the two aspartic-rich region motifs (Motif 1: DDVYD; motif 2: NDSATHSDE) necessary for Mg^{2+} coordination are shaded in orange. The RXR motif is shaded in pink. The four aminoacids of the IspS diagnostic tetrad (F_{310}, S_{418}, F_{457}, S_{477}), all relevant for enzymatic activity, are shaded in red.
